# Psychometric evaluation of the Swedish version of ages and stages questionnaire social-emotional: second edition for parents of children 18 months of age

**DOI:** 10.1186/s40359-024-01996-z

**Published:** 2024-10-17

**Authors:** Anna Edenius, Malin Bergström, Lene Lindberg, Kersti Bergqvist, Anna Fröjlinger, Pia Enebrink, Johan Åhlén

**Affiliations:** 1 Department of Medicine, Karolinska Institutet, Stockholm, Sweden; 2 Center for Epidemiology and Community Medicine, Stockholm County, Sweden; 3 Department of Global Public Health, Karolinska Institutet, Stockholm, Sweden; 4 The Child Health Services unit, Stockholm, Sweden; 5 Department of Clinical Neuroscience, Karolinska Institutet, Stockholm, Sweden

**Keywords:** Child assessment, Children, Developmental screening, Infants, Mental health, Reliability, Risk, Socioemotional development, Validity

## Abstract

**Background:**

Social and emotional development are important aspects of young children’s well-being but can be difficult to assess during the first years of life. The Ages and Stages Questionnaire: Social-Emotional Second Edition (ASQ:SE-2) is a parent-rated assessment tool for child socioemotional development between 1 and 72 months of age. In this study, we examined the psychometric properties of this instrument in 18-month-old Swedish children.

**Methods:**

Data from 586 Swedish-speaking parents of 18-month-old children were included. In addition to the ASQ:SE-2, parents also completed the Social-Emotional Assessment/Evaluation Measure (SEAM), and the child’s socioemotional development was assessed by a nurse at the Child Health Services. We used exploratory factor analysis and Rasch methodology to explore dimensionality and item properties of the ASQ:SE-2. Furthermore, we used Pearson and Spearman rank correlations to study associations with the SEAM and the nurse assessment.

**Results:**

An exploratory factor analysis suggested a one-factor model for the ASQ:SE-2 items. However, several items showed weak factor loadings, and a final scale including 18 of the original 29 items was further explored. The Rasch analysis revealed problems with targeting, and the final scale showed acceptable reliability only in the 22% with the highest levels of socioemotional difficulties. The total score of the final version showed a strong association with the parent-rated SEAM but a weak association with the nurse observations. We labeled the reduced 18-item scale *Social Interaction*.

**Conclusions:**

The results of this study highlight that the original Swedish version of the ASQ:SE-2 for 18-month-old children may not be an adequate tool for assessing social and emotional competencies in a normal population since acceptable reliability was reached only in children with the greatest difficulties (above the 78th percentile). In conclusion, the suggested 18-item version works best either as a screening instrument for problems with social interaction or as a continuous measure of such problems in children with high levels of social interaction difficulties.

**Supplementary Information:**

The online version contains supplementary material available at 10.1186/s40359-024-01996-z.

## Background

The first years of life are crucial for human development, as they influence well-being and mental and physical health throughout the lifespan [1, 2]. Children’s social and emotional abilities influence other aspects of their development, such as communicative and cognitive competence, as well as the ability to establish meaningful and lasting relationships [1, 3, 4]. A common description of social and emotional competence for children 1–3 years of age implies the ability to understand one’s own feelings and needs as well as how to interact with and what to expect from others [3]. Early social and emotional difficulties may have an impact on later development and are hence important to recognize to prevent subsequent negative development [1, 5].

Despite the importance of assessing social and emotional abilities and delays in young children, this may be challenging. Their development is fast and diverse, and clear definitions and conceptualizations are lacking [4]. A literature review identified 75 instruments for assessing socioemotional development in young children and suggested four subdomains: social competence, emotional competence, behavior problems and self-regulations [6]. Six out of the 75 instruments were identified as suitable candidates for wider application with children from birth to age five, as they demonstrated the majority of ten key features characteristic of robust assessments [6].

In Sweden, Child health services (CHS) are publicly financed and reach almost all children from birth to 5 years of age [7]. The CHS provides health surveillance, distributes the vaccination program and offers parental support, and conducted by nurses. CHS nurses are hence specialized in assessing various aspects of development in young children. During infancy the family’s contact with the CHS nurse is intense, with about ten scheduled individual appointments during the child’s first 18 months, according to the national guidelines. After child aged 10 months the support from the CHS nurse gradually gets sparser, with regular appointments at 12 and 18 months, and the next when the child is 3 years old [8]. From an international perspective, both the participation rate and the equal access to the Swedish CHS are high [9]. In the Stockholm region, which includes one-fourth of Sweden’s 0–5-year-olds, child behavior and socioemotional development are assessed in a structured way at ages 3 and 4. For the younger age groups, such structured assessment has not yet been implemented. A study of 3-year-olds has shown that behavioral problems and social interaction issues are the most common concerns presented by parents [10] (Hjern et al., 2024). When the support provided by the CHS nurse is insufficient, children and parents are referred to CHS psychologists or manual-based parenting support programs. Despite these favorable conditions for providing early support and detecting children at risk, there is no gold standard for assessing social and emotional development in children under three years of age [6, 11]. The Ages and Stages Questionnaire: Social-Emotional Second Edition (ASQ:SE-2) is an internationally well-used parent-rated assessment tool for assessing socioemotional development in children 1–72 months of age [12, 13] see Appendix 1.

To summarize, behavioral problems and social interaction issues are main concerns for Swedish parents during the CHS period [10]. Professionals in the CHS hence need structured, validated tools to assess social and emotional development in the youngest children. Such instruments are currently lacking in Sweden for children aged 0–3 years. Therefore, this study may contribute to the knowledge regarding the early identification of social and emotional difficulties, as well as the lack of validated assessment tools for children under 3 years of age.

### Previous research on the ASQ:SE

The psychometric properties of the ASQ:SE have been studied worldwide, with heterogeneous results regarding reliability and validity [11, 12, 14–18]. Higher reliability has been reported for English versions than for translated versions [11, 14]. Internal consistency has often been found to be acceptable-to-good but with lower reliability coefficients for younger age groups [11, 12]. A study of the Spanish version of the ASQ:SE in Uruguay reported a floor effect in most age ranges [14]. A few studies have analyzed the factorial structure of the ASQ:SE [14, 15, 19]. According to these analyses, a two-dimensional model showed the best fit for the 18-month questionnaire.

## Methods

### Aim, design and setting

The aim of this study was to analyze the psychometric properties (dimensionality, item fit, targeting, reliability and correlation with other measures) of the Swedish 18-month version (age span 15–20 months) of ASQ:SE-2 among parents in the Stockholm region. The participating parents completed the ASQ:SE-2 online together with questions on sociodemographic characteristics and another instrument, the Social-Emotional Assessment/Evaluation Measure (SEAM). Children’s social and emotional development was also assessed by a nurse at the CHS during the regular 18-month visit.

### Procedure and participants

All 125 CHS units in the Stockholm region with approximately 500 female nurses were introduced to the project. The inclusion criterion for nurses’ participation was at least two years of clinical experience within the CHS. A total of 46 nurses were included and received two half-day training seminars on social and emotional development at the age of 18 months and on how to assess this development with the study-specific nurse assessment protocol. From October 2021–December 2022, they invited all Swedish-speaking parents to 18-month-old children at their CHS units to participate in the study. Eligible parents received written information about the study and a link to a study-specific digital platform. On this platform, they could confirm their consent and fill out the questionnaire a few weeks before their ordinary 18-month visit at the CHS. Two reminders were sent to parents who consented to participate but did not complete the questionnaire. A total of 706 parents were included.

The nurses could not access parents’ responses, but parents were encouraged to raise any concerns about their children with the nurse. During the visit, the CHS nurse performed a structured assessment of the child’s social and emotional development according to the study-specific protocol (see Appendix 2). If the nurse was concerned about something in the child’s development, she shared her observations with the parents. Ethical approval (registration number 2021–02265) and informed consent from all participating parents and nurses were obtained.

### Measures

#### Sociodemographic questions

The parental questionnaires contained sociodemographic questions on child sex, siblings, family structure, parental age, educational level and country of birth. Questions about the relevance and feasibility of the questionnaire were also included.

#### Ages and stages questionnaire: social–emotional, second edition (ASQ:SE-2) Swedish version

The ASQ:SE-2 is a parent-completed assessment of children’s social and emotional development and consists of eight age-specific questionnaires from 1 to 72 months [12, 13]. Seven behavior areas are measured: *self-regulation*,* compliance*,* communication*,* adaptive functioning*,* autonomy*,* affect* and *interaction with people*. The items are scored as “most of the time” (0 points), “sometimes” (5 points) or “rarely or never” (10 points).

We used a previous Swedish translation of the first version of the ASQ:SE 18-month questionnaire [20], which followed recommended principles for translation [21]. The five items (numbers 26, 28, 27, 29, and 30) that were added to the revised second version (ASQ:SE-2) were translated by our research group according to the guidelines for cultural and linguistic adaptation of the ASE:SE-2 [13].

The original version of the ASQ:SE-2 18-month questionnaire consists of 34 items. During 2021, we conducted a pilot study with five experienced CHS nurses to assess the feasibility and acceptability of the instrument. The nurses asked parents to complete the ASQ:SE-2 during their regular 18-month visit at CHS, while the nurses noted parents’ comments and opinions about the questions, according to the “think aloud” interview method [22]. Based on these interviews, three items were excluded (item 5 “Is your child’s body relaxed?”, item 8 “Does your child stiffen and arch his back when picked up?” and item 15 “Does your child sleep at least 10 hours in a 24-hour period?”) since parents and CHS nurses perceived them inexplicit and difficult to answer. For item number 7, “When upset, can your child calm down within 15 minutes?”, the wording “…if the child gets comforted?” was added to the question. Items 33 and 34 were excluded from our analyses because of their free text responses. The adjusted questionnaire used in this study hence consists of 29 items (see Appendix 1).

#### Social-emotional assessment/evaluation measure (SEAM)

SEAM is a strength-based tool aimed at assessing young children’s social and emotional skills and competences [23]. The toddler version of the questionnaire (18–36 months) consists of 35 items scored on a scale of 0–3 as “Very True”, “Somewhat true”, “Rarely true” and “Not true”. High scores indicate high functioning. Every item is followed by an example such as “The child talks about other children’s feelings. For example, gives a pacifier or a toy to a child who is sad”. The items are summarized into ten benchmarks: (1) *child participates in healthy interactions*, including four items; (2) *child expresses a range of emotions*, including four items; (3) *child regulates socio-emotional responses*, including three items; (4) *child begins to show empathy for other responses*, including three items; (5) *child attends to and engages with other responses*, including five items; (6) *child demonstrates independence*, including three items; (7) *child displays a positive self-image*, including three items; (8) *child regulates activity level*, including four items; (9) *child cooperates with daily routines and requests*, including two items; and (10) *child shows a range of adaptive skills*, including four items.

In a Danish study, a Rasch analysis revealed that the 10 benchmarks can be combined into two unidimensional indexes: Empathy and Self-regulation & Cooperation [24]. Using a Monte-Carlo estimation of the reliability coefficients [25] the authors found good reliability for boys and girls aged 18–23 months. The Danish version of SEAM was adapted in consultation with SEAM’s lead author, Jane Squires, and the process followed recommended translation principles [26]. The Swedish version of the SEAM in this study was translated from the Danish version with minor adjustments, as the languages are very similar.

#### Assessment protocol for CHS nurses

For validation purposes, we developed a study-specific protocol for CHS nurses’ assessments of children’s social and emotional development during the regular 18-month CHS visit (see Appendix 2). Based on the behavior areas of the ASQ:SE-2 subscales; *self-regulation*, *compliance*,* communication*,* adaptive functioning*, *autonomy*,* affect* and *interaction with people*, we constructed 22 questions. The nurses answered on a scale ranging from 0 to 5 points, from “Not at all”=0 points to “Most of the time”=5 points, based on what they observed during the visit. For example, to correspond to the ASQ:SE-2 question “Does your child cry, scream or have tantrums for long periods of time?”, we asked the nurses to assess “Does the child cry, scream or have tantrums during the visit?”. To capture the scope of item 10 in the ASQ:SE-2 “Is your child interested in things around her, such as people, toys, and foods?”, we asked the nurses to assess “Is the child interested in you during the visit?”. Unfortunately, we did not have data to calculate the inter-rater reliability for the CHS nurses’ assessments. This would have required multiple nurses to assess the same children, for example using videorecorded sessions, which was not feasible within the healthcare setting.

### Data analysis

All the statistical analyses were performed using the “psych” and “RISEkbmRasch” packages in *R*. In our analyses of the psychometric properties of the ASQ:SE-2, we included the 586 participants with complete questionnaires. We chose not to impute data to avoid potential biases that imputation methods could introduce in psychometric analysis, such as overestimating the scale’s quality [27, 28]. Internal attrition analyses were conducted to compare sociodemographic variables between cases with complete data and those excluded. Frequencies were analyzed by Chi-square test and means and standard deviations were analyzed by t-test.

### Step 1 – dimensionality

To explore dimensionality of the ASQ:SE-2, we used exploratory factor analysis (EFA) together with three methods to estimate the underlying number of factors, i.e., a visual inspection of the scree plot, the Very Simple Structure, VSS [29], and the Velicer´s minimum average partial test, MAP [30]. EFAs were based on polychoric correlations to accurately generate factor solutions when using ordinal data [31]. We used a maximum likelihood factoring method and the oblimin rotation to extract and interpret the results, as suggested by Costello et al. [32].

### Step 2 – item reduction

Given the best model (based on the EFAs), we reduced items with low factor loadings (< 0.40) [33] because of their low association to the latent construct. Further, data should be in accordance with a Rasch model to justify a summation of scores [34]. Therefore, we explored the remaining items in a polytomous Rasch model (using the Partial Credit Model) and examined (1) the correlations of Rasch residuals (2), item fit, and (3) targeting. Strong correlations of Rasch residuals that a response on one item is associated to the response of another item, which causes bias in the summation of scores and inflated reliability indices [35]. Strong residual correlation was defined as 0.2 above the mean item residual correlation [36]. The mean square fit statistics are used to explore if an item introduces noise (i.e., a misfitted item) or is redundant to the scale (i.e., an overfitted item). A mean square above 1.5 indicates noise, and a mean square below 0.5 indicates redundancy [37]. Targeting of the items explores if the response category thresholds match the distribution of the population of the latent construct. Gaps in targeting for certain areas generate low measurement reliability for the population, as there are no (or limited) thresholds to differentiate between individuals.

### Step 3 – correlations with other measures

Furthermore, we explored Pearson correlations between total scores on the final version of the ASQ:SE-2 and the SEAM total score and subscales scores. To gain more information on what the final set of the ASQ:SE-2 items measured, we also explored Spearman rank correlations between total scores on the final version of the ASQ:SE-2 and the study-specific protocol with CHS nurses’ ratings.

### Step 4 – reliability

Reliability was examined with the Test Information Function (TIF) curve, that illustrates reliability for the different levels of the latent construct [35]. TIF values of 3.33 correspond to a reliability coefficient of 0.7, typically considered the lower bound for acceptable reliability [38].

## Results

### Sociodemographic characteristics

Of the 706 parents of 18-month-old children who answered the online questionnaire, 586 (83%) had complete data on the ASQ:SE-2 and were included in the analyses. More than half of the parents assessed their children’s socioemotional development together as a couple. The average parental age was 35 years for mothers and 37 years for fathers, 80% of the mothers and almost 70% of the fathers had a university education, and the majority, almost 80% of the parents, were born in Sweden. Among the children, slightly more boys than girls were included, and almost half of the children were only children. Most children lived with both parents, while a few percent lived mostly or only with one parent (see Table 1).

**Table 1:** Sociodemographic characteristics of parents and children, shown in frequencies, percentages, means, and standard deviations (*N* = 586)

	Frequency (*n*)	Percent (%)	Mean (SD)
**Child sex**			
Boys	314	54%	
Girls	270	46%	
Missing	2	< 1%	
**Siblings**			
Only child	285	49%	
Younger	8	1%	
Older	283	48%	
Both younger and older	9	1%	
Missing	1	< 1%	
**Living arrangements**			
With both parents	569	97%	
Mostly or only with one parent	17	3%	
**Respondent**			
Fathers	67	11%	
Mothers	186	32%	
Both father and mother	327	56%	
Parents with same sex	6	1%	
**Parental age**			
Mothers			34.7 (4.2)
Fathers			36.6 (4.7)
Mothers’ educational level*			
Primary/Secondary school	103	20%	
University	421	80%	
Missing	1	< 1%	
Fathers’ educational level*			
Primary/Secondary school	118	30%	
University	272	69%	
Missing	4	1%	
**Mothers’ country of birth**			
Sweden	410	78%	
Abroad	80	15%	
Missing	35	7%	
**Fathers’ country of birth**			
Sweden	314	80%	
Abroad	63	16%	
Missing	17	4%	

Notes: *The highest level of education of the parents

In the attrition analyses, we found negligible differences between those included in our study sample and those excluded, except in regard to respondents. In the study sample, significantly more parents than in the excluded group had completed the questionnaire together (see Appendix 3).

### Dimensionality

A visual inspection of the scree plot indicated one or two factors (see Supplementary Figure S1). The VSS and the MAP indices also suggested one or two factors respectively. In a one-factor solution 18 of the 29 items (62%) showed strong factor loadings (> 0.40). In a two-factor solution only 14 of the 29 items (48%) showed strong factor loadings on either of the two factors, and the second factor included only five items. See Table 2 for the one- and two-factor EFAs. As the two-factor solution did not present a better model, we chose the one-factor model for further analysis.

**Table 2:** Exploratory factor analysis of the total set of 29 items of the ASQ:SE-2 as one- and two-factor models

Item nr	One-factor solution	Two-factor solution	
	Factor 1	Factor 1	Factor 2
1	0.61	0.50	
2	0.48		0.56
3	0.45		
4			
6	0.47		
7	0.48		0.68
9			0.76
10	0.79	0.72	
11			
12	0.51		
13			
14	0.49		
16	0.63	0.66	
17			
18	0.45	0.53	
19	0.58	0.60	
20	0.54		
21			
22			
23			
24	0.46		0.41
25			0.48
26	0.53	0.68	
27	0.59	0.73	
28	0.54	0.65	
29			
30	0.67	0.66	
31	0.43		
32			
Explained variance	21%	17%	11%

Notes: Loadings below 0.40 have been removed from the table

### Item reduction

We excluded the eleven items with low loadings according to the one-factor EFA (see Table 2). The excluded items were 4, 9, 11, 13, 17, 21–23, 25, 29 and 32. Items 9, 11, 13, and 25 are from the subscale *self-regulation*, items 17, 23 and 29 are from the subscale *adaptive functioning*, items 4 and 22 are from the subscale *interaction with people* and item 21 is from the subscale *autonomy*. The single-item subscale *autonomy* was hence entirely deleted, whereas only one item was left after the reduction in the two subscales *self-regulation* and *adaptive functioning* (see Appendix 1).

To summarize, the retained items came from the subscales *self-regulation*; one item (7), *compliance*; one item (19), *adaptive functioning*; one item (12), *affect*; two items (6 and 10), *communication* seven items (1, 16, 18, 26–28, 30) and *interaction with people* with five items (2, 3, 14, 20 and 24). Furthermore, item 31, which is not included in any subscale (“Has anyone shared concerns about your child’s behaviors?”), remained in the final dataset (see Appendix 1). Owing to the selection of items in the remaining data, we labeled the latent construct in this one-factor scale *Social Interaction.*

In further analyses, only two pairs of items showed high residual correlations (items 1 and 30 and items 26 and 27). Both correlations were marginally higher than 0.2 above the mean residual correlation (see Supplementary Table S1). When exploring item fit, no item indicated a misfit (i.e., no item above 1.5). However, one item (item 10) indicated redundancy (see Supplementary Table S2). Based on the low residual correlations and acceptable item fit, we decided to retain all 18 items in the final version of the ASQ:SE-2. When looking at targeting, response category thresholds were generally higher than the main part of the population’s level on the latent construct (see Supplementary Figure S2). In summary, all thresholds were located above the average level of the latent construct, indicating large problems in differentiating between individuals with low and medium levels of the latent construct.

### Reliability

**Fig. 1:**
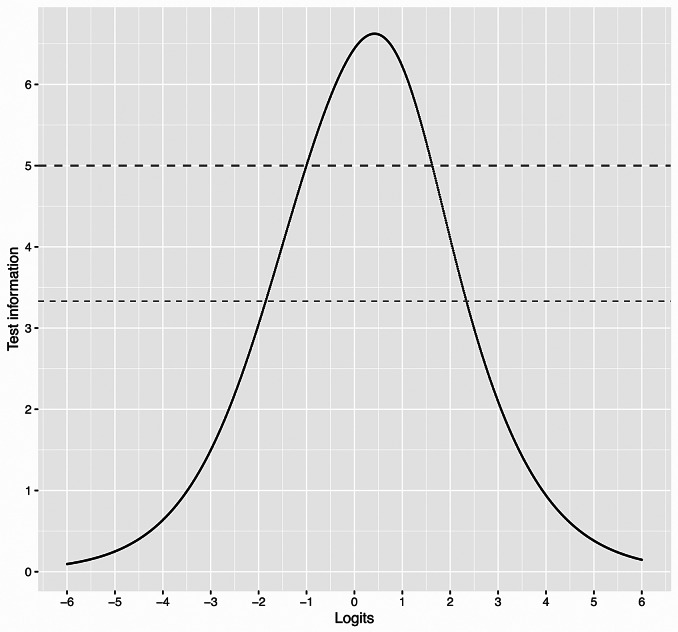
Test Information Function (reliability). Note: Test Information 3.33 (equals reliability of 0.7) is reached between − 1.85 and 2.34 logits, where 22.2% of the participants are located

According to the TIF (Test Information Function) curve, the final 18-item scale only showed acceptable reliability in 22% of the study sample with the highest levels of the latent construct (see Fig. 1).

### Correlations with other measures

#### ASQ:SE-2 and SEAM

As hypothesized, the total score of the final version of the ASQ:SE-2 showed a strong negative (as hypothesized) correlation with the total score of the SEAM (*r* = − .67). When examining the two-index split of the SEAM, the ASQ:SE-2 had a somewhat stronger negative correlation with the Empathy Index (*r* = − .63) compared with the Self-regulation & Cooperation Index (*r* = − .58). Finally, regarding the ten benchmarks of the SEAM, the ASQ:SE-2 showed the strongest negative correlation with benchmark 5, *child shares attention and engages with others*’ responses” (*r* =-.55), and the weakest negative correlation with benchmark 3, “*child regulates socio-emotional responses*” (*r* =-.32), see Table 3.

**Table 3:** Pearson correlations between the SEAM and the ASQ:SE-2 total scores

SEAM	ASQ:SE-2 18 total score
SEAM benchmark 1	-0.51***
SEAM benchmark 2	-0.40***
SEAM benchmark 3	-0.31***
SEAM benchmark 4	-0.44***
SEAM benchmark 5	-0.55***
SEAM benchmark 6	-0.36***
SEAM benchmark 7	-0.43***
SEAM benchmark 8	-0.44***
SEAM benchmark 9	-0.45***
SEAM benchmark 10	-0.41***
SEAM Empathy Index	-0.63***
SEAM Self-regulation & Cooperation Index	-0.59***
SEAM Total scores	-0.67***

* p < .05; ** *p* < .01; *** *p* < .001

#### ASQ:SE-2 and CHS nurse ratings

We conducted analyses on the 14 items in the CHS nurse protocol that corresponded to the items in the 18-item version of the ASQ:SE-2 (see Appendix 2). The total score of the final version of the ASQ:SE-2 showed Spearman Rank correlations between 0.00 and 0.23 with CHS nurse ratings. The strongest correlations were found between the ASQ:SE-2 and the nurse assessments “Does the child seem to have sleeping, eating, or elimination problems?”, “Do you have any concerns about the child?” and “Does the child follow simple directions? For example, bringing objects when asked?”. The weakest correlations were found with the items “Does the child have difficulties letting go of the parent in a way that affects the completion of the visit?”, “Does the child laugh or smile when you play together?” and “Does the child seem to like being hugged or cuddled by the parent?”, see Table 4.

**Table 4:** Spearman rank correlations between CHS nurse assessments and ASQ:SE-2 18 total scores

CHS nurse assessments	Spearman Rank correlation with the ASQ:SE-2 total score of 18 items
Does the child look at you when you talk to them?	0.11*
Does the child laugh or smile when you play together?	0.02
Does the child seem to like being hugged or cuddled by the parent?	0.05
Does the child have difficulties letting go of the parent in a way that affects the completion of the visit?	0.00
Does the child seem to be interested in other children or people?	0.14**
Does the child seem to have sleeping, eating, or elimination problems?	0.23***
When you point at something, does the child look in the direction you are pointing?	0.10*
Does the child follow simple directions? For example, bringing objects when asked?	0.18***
Does the child seem to be interested in you during the visit?	0.06
Does the child try to show you things by pointing at them and looking back at you?	0.07
Does the child make sounds or use words or gestures to let you or the parent know they wants something (for example, by reaching)?	0.09*
Does the child play with objects by pretending? For example, does the child pretend to talk on the phone, feed a doll or a toy animal?	0.12**
Does the child respond to their name? For example, by turning their head and look at you?	0.14***
Do you have any concerns about the child?	0.21***

* p < .05; ** *p* < .01; *** *p* < .001

## Discussion

In this study, we examined the psychometric properties of the widely used parent-rated assessment tool ASQ:SE-2 in 586 Swedish parents of 18-month-old infants. The analyses were conducted in four steps: (1) dimensionality (2), item reduction (3), reliability and (4) correlation with other measures. Few previous studies on the psychometric properties of the ASQ:SE have included analyses of the factorial structure. To the best of our knowledge, none of these studies have suggested a one-factor model as the best fit for the 18-month version (age span 15–20 months) but instead two distinct factors [14, 15, 19]. In the one-factor model suggested here, we included only 18 items of the original 29. Twelve of the 18 remaining items were found in the subscales *communication* and *interaction with people*, and we labeled the construct *Social Interaction*. The 11 excluded items did not fit any additional factors/dimensions. Many of these items were from the self-regulation subscale, which means this aspect of socio-emotional development was nearly entirely removed. Clearly, this results in a narrowing of the construct and a reduction in content coverage [39]. However, it is essential to demonstrate that items collectively measure a single latent variable. While other important aspects of socio-emotional development may exist, they should be considered as separate unidimensional scales [30]. Unfortunately, since we did not find evidence that the excluded items formed a cohesive subscale, we did not further evaluate them, and aspects beyond social interaction were not explored.

In the analyses, we also identified a strong negative correlation with the SEAM measure but weak correlations with the nurse assessments. We also found that the 18-item version of the ASQ:SE-2 reliably could assess only children with a high degree of social interaction difficulties. For the majority of children (with less difficulties) reliability was low. This result stems from a deficiency in response categories capable of differentiating between children with lower problem levels, resulting in a floor effect in the score distribution. Similar findings have been observed with the ASQ:SE in other samples [14]. This poses a challenge for measuring lower levels of the construct. To address this, adding new items with lower difficulty or adjusting and increasing the number of response categories could be considered.

The strong correlation between the ASQ:SE-2 and the SEAM indicates that there is an overlap, which means that the two tools measure similar latent constructs. Children are born with a repertoire of social behaviors but with no ability to regulate strong emotions on their own [40]. At 18 months of age, children are still in crucial need of emotion regulation from a responding and caring surrounding [41]. When analyzing the 18-item ASQ:SE-2, we found that the questions aimed to capture how the child relates, engages, and responds to others. From this perspective, a latent construct labeled *Social Interaction* is coherent and in line with young children’s development. It is also consistent with the strong correlation with SEAM and the strongest correlation with benchmark 5: *child shares attention and engages with others’ responses* (see Table 3). The weak or nonexistent correlations between the ASQ:SE-2 and the nurse ratings may be explained by the different informants and settings and is well known from previous research [42]. Parents assessed their child’s overall functioning in daily life, whereas the nurse assessed her observation during the CHS visit. Parents’ structured assessment may hence contribute to the professional observations by adding information on child difficulties that are not apparent during visits at the CHS.

In summary, the results of this study indicate that this instrument may not be suitable for collecting information on social and emotional development in 18-month-old children in a normal Swedish population. Its reliability was acceptable only at higher levels of the latent construct. Therefore, the ASQ:SE-2 might be used as a screening instrument to identify indicated, more severe *Social Interaction* difficulties, or as a continuous measure but limited to populations with high levels of these difficulties.

### Further research

In the present study, we analyzed the psychometric properties of the 18-month questionnaire only, and therefore we cannot say anything about the properties of the other age spans of ASQ:SE-2. Hence, further research is needed to obtain a full understanding of how this instrument works at different ages in a Swedish setting. Establishing new norms and cut-offs would be crucial aspects of such studies.

Previous research has revealed the lack of clear definitions and conceptualizations of children’s social and emotional abilities [4]. Nevertheless, owing to the importance of these abilities for overall development, there is an urgent need of proceeding the development of reliable and valid instruments for early detection of social and emotional delays and disabilities.

It would be interesting to follow children with more indicated difficulties over time to gain a deeper understanding of the dimensions assessed by this instrument. Such studies could shed light on the developmental trajectories of these children over time and help identify the specific support that these families need to provide the optimal conditions for positive and resilient growth in this group of children.

### Strengths and limitations

To the best of our knowledge, most of the validation studies on the ASQ:SE are on mothers as respondents. This study benefits from a substantial sample size and a narrow age range, as well as from the comparably high frequency of participating fathers and parents who have assessed their child’s development together. We have hence been able to include the perspective of both parents, which may provide a more nuanced picture of the child’s development.

Attrition analyses revealed only negligible differences between the study sample and the excluded cases (Appendix 3), but parents in the study group had a higher educational level compared with the general Swedish population [43]. The proportion of parents born outside of Sweden was, however, similar to that of the general population.

One limitation of our study is the potential dependency in the nurse ratings. Given that multiple nurses assessed the same set of children, this clustering could affect the reliability of the correlation estimates between nurse assessments and the ASQ:SE-2. Specifically, the dependency might inflate standard errors and lead to more frequent findings of statistical significance than would be the case with truly independent observations. While we acknowledge this limitation, our analysis was primarily focused on identifying the relative associations between individual items and the ASQ:SE-2, rather than on testing the significance of these associations.

## Conclusions

This study suggests a reduced scale (18 items) as an overall measure of social interaction problems. The results highlight that the Swedish version of the ASQ:SE-2 for 18-month-old children may not be an adequate tool for assessing social and emotional competencies in a normal population. Hence, the suggested 18-item version works best either as a screening instrument for problems with social interaction or as a continuous measure of such problems in a population with high levels of social interaction difficulties.

## Electronic supplementary material

Below is the link to the electronic supplementary material.

Supplementary Material 1

Supplementary Material 2

Supplementary Material 3

Supplementary Material 4

## Data Availability

Due to the ethical approval (registration number 2021-02265) which states that the information from participating parents and CHS nurses should respect anonymity, and only be accessed by the research group and stored on secure platforms at Karolinska Institutet. Data are only available from the corresponding author upon a reasonable request.

## Appendix 1. Included and excluded (in gray) items in the Swedish version of ASQ:SE-2 for 18-month-old children

We excluded the eleven items with low loadings according to the one-factor EFA (Table 2).

## Appendix 3. Internal attrition analyses on sociodemographic characteristics between study sample (*N* = 586) and those excluded (*N* = 120). Shown in percentages, means, standard deviations and test difference

**Table:**

	Study sample *n* (%)	Attrition *n* (%)	Test difference^a^Chi-square and t-test
**Child gender**			
Boys	314 (54%)	69 (57%)	
Girls	270 (46%)	49 (41%)	0.40
Missing^b^	2 (< 1%)	2 (< 1%)	
**Siblings**			
Only child	285 (49%)	51 (43%)	
With siblings	300 (51%)	64 (53%)	0.45
Missing^b^	1 (< 1%)	5 (4%)	
**Living arrangements**			
With both parents	569 (97%)	111 (92%)	
Other living arrangements	17 (3%)	6 (5%)	0.34
Missing^b^	…	3 (3%)	
**Respondent**			
Fathers	67 (11%)	16 (13%)	
Mothers	186 (32%)	51 (42%)	
Parents together	333 (57%)	52 (43%)	0.03*
**Parental age in years (means**,** SD)**			
Mothers	34.7 (4.2)	35.4 (4.1)	0.11
Fathers	36.6 (4.7)	37.7 (6.3)	0.18
**Mothers’ educational level** ^c^			
Primary or Secondary	103 (20%)	17 (16%)	
University	421(80%)	83 (81%)	0.63
Missing^b^	1 (< 1%)	3 (3%)	
**Fathers’ educational level** ^c^			
Primary or secondary	118 (30%)	16 (24%)	
University	272 (69%)	49 (72%)	0.44
Missing^b^	4 (1%)	3 (4%)	
**Mothers’ country of birth**			
Sweden	410 (78%)	65 (63%)	
Abroad	80 (15%)	21 (20%)	0.10
Missing^b^	35 (7%)	17 (17%)	
**Fathers’ country of birth**			
Sweden	314 (80%)	51 (75%)	
Abroad	63 (16%)	9 (13%)	0.89
Missing^b^	17 (4%)	8 (12%)	

Notes: ^a^Frequencies analyzed by Chi-square test, means and standard deviations by t-test;

^b^ Excluded from difference test; ^c^The highest level of education of the parents

* *p* < .05; ** *p* < .01; *** *p* < .001

## Abbreviations

ASQ: Ages and Stages Questionnaire
SE-2: Social–Emotional, Second Edition
CHS: Child health services
EFA: Exploratory Factor Analysis
MAP: the Velicer´s minimum average partial test
SEAM: Social-Emotional Assessment/Evaluation Measure
VSS: the Very Simple Structure
TIF: the Test Information Function curve
