# Supplementary material for: Psychometric evaluation of the Swedish version of ages and stages questionnaire social-emotional: second edition for parents of children 18 months of age
Source: BMC Psychol. 2024 Oct 17;12:564. doi: 10.1186/s40359-024-01996-z (PMC11487771; doi:10.1186/s40359-024-01996-z)
Supplement: Supplementary file 1 — Supplementary Material 1 [file 40359_2024_1996_MOESM1_ESM.docx]

***Supplementary Figure S1: Scree plot of the total 29 items***
