# Supplementary material for: Psychometric evaluation of the Swedish version of ages and stages questionnaire social-emotional: second edition for parents of children 18 months of age
Source: BMC Psychol. 2024 Oct 17;12:564. doi: 10.1186/s40359-024-01996-z (PMC11487771; doi:10.1186/s40359-024-01996-z)
Supplement: Supplementary file 2 — Supplementary Material 2 [file 40359_2024_1996_MOESM2_ESM.docx]

***Supplementary Figure S2: Persons and threshold locations***

Location (logit scale)

*Person location average: -2.84 (SD 1.15), Item threshold location average: 0.37 (SD 1.2).*
