# Supplementary material for: Psychometric evaluation of the Swedish version of ages and stages questionnaire social-emotional: second edition for parents of children 18 months of age
Source: BMC Psychol. 2024 Oct 17;12:564. doi: 10.1186/s40359-024-01996-z (PMC11487771; doi:10.1186/s40359-024-01996-z)
Supplement: Supplementary file 3 — Supplementary Material 3 [file 40359_2024_1996_MOESM3_ESM.docx]

***Supplementary Table S1: Residual correlation of Rasch residuals***

|  | ASQ1 | ASQ2 | ASQ3 | ASQ6 | ASQ7 | ASQ10 | ASQ12 | ASQ14 | ASQ16 | ASQ18 | ASQ19 | ASQ20 | ASQ24 | ASQ26 | ASQ27 | ASQ28 | ASQ30 |
| --- | --- | --- | --- | --- | --- | --- | --- | --- | --- | --- | --- | --- | --- | --- | --- | --- | --- |
|  |  |  |  |  |  |  |  |  |  |  |  |  |  |  |  |  |  |
| ASQ2 | -.04 |  |  |  |  |  |  |  |  |  |  |  |  |  |  |  |  |
| ASQ3 | .00 | -.01 |  |  |  |  |  |  |  |  |  |  |  |  |  |  |  |
| ASQ6 | .02 | -.05 | .08 |  |  |  |  |  |  |  |  |  |  |  |  |  |  |
| ASQ7 | -.01 | .12 | .01 | -.01 |  |  |  |  |  |  |  |  |  |  |  |  |  |
| ASQ10 | .07 | -.03 | -.01 | .-03 | -.10 |  |  |  |  |  |  |  |  |  |  |  |  |
| ASQ12 | .03 | .15 | -.11 | -.12 | .00 | .07 |  |  |  |  |  |  |  |  |  |  |  |
| ASQ14 | -.02 | -.04 | -.03 | .03 | -.01 | .02 | 0.11 |  |  |  |  |  |  |  |  |  |  |
| ASQ16 | -.04 | -.11 | -.03 | -.12 | -.11 | .07 | -.04 | -.08 |  |  |  |  |  |  |  |  |  |
| ASQ18 | -.10 | -.04 | -.06 | -.04 | -.08 | -.10 | -.22 | -.23 | .00 |  |  |  |  |  |  |  |  |
| ASQ19 | .08 | -.09 | -.10 | -.14 | -.04 | -.08 | -.13 | -.07 | .06 | -.05 |  |  |  |  |  |  |  |
| ASQ20 | -.03 | -.04 | .07 | .00 | .04 | .16 | -.08 | .00 | -.01 | -.03 | -.09 |  |  |  |  |  |  |
| ASQ24 | -.06 | .02 | .09 | -.05 | .09 | -.02 | -.06 | .04 | -.06 | -.10 | -.12 | .09 |  |  |  |  |  |
| ASQ26 | -.13 | -.06 | -.05 | -.07 | -.09 | .03 | -.22 | -.19 | -.01 | .00 | -.03 | -.01 | -.08 |  |  |  |  |
| ASQ27 | -.04 | -.06 | .01 | .-01 | -.09 | .07 | -.03 | -.07 | .03 | .04 | -.01 | -.08 | -.20 | **.20** |  |  |  |
| ASQ28 | -.10 | -.11 | -.01 | -.17 | -.03 | .11 | -.21 | -.16 | -.01 | .00 | -.06 | -.02 | -.14 | .08 | .01 |  |  |
| ASQ30 | **.23** | -.13 | -.04 | .00 | -.05 | -.01 | -.10 | -.05 | .04 | -.12 | .14 | -.04 | -.07 | -.03 | .08 | -.05 |  |
| ASQ31 | -.07 | .06 | -.04 | -.15 | -.05 | -.05 | .09 | -.11 | -.08 | -.04 | .01 | -.06 | .08 | -.04 | -.09 | -.11 | -.11 |

*Note:* Relative cut-off value (in bold) is 0.168, which is 0.2 above the average correlation.
