# Supplementary material for: Psychometric evaluation of the Swedish version of ages and stages questionnaire social-emotional: second edition for parents of children 18 months of age
Source: BMC Psychol. 2024 Oct 17;12:564. doi: 10.1186/s40359-024-01996-z (PMC11487771; doi:10.1186/s40359-024-01996-z)
Supplement: Supplementary file 4 — Supplementary Material 4 [file 40359_2024_1996_MOESM4_ESM.docx]

***Supplementary Table S2: Mean square item fit***

|  | *Outfit* | *Infit* |
| --- | --- | --- |
| ASQ1 | 0.66 | 0.91 |
| ASQ2 | 0.96 | 0.97 |
| ASQ3 | 0.95 | 0.97 |
| ASQ6 | 1.14 | 1.06 |
| ASQ7 | 0.90 | 0.98 |
| ASQ10 | **0.27** | 0.86 |
| ASQ12 | 1.15 | 1.05 |
| ASQ14 | 1.04 | 1.02 |
| ASQ16 | 0.83 | 0.90 |
| ASQ18 | 0.95 | 0.97 |
| ASQ19 | 0.86 | 0.87 |
| ASQ20 | 0.78 | 0.94 |
| ASQ24 | 0.96 | 1.01 |
| ASQ26 | 0.85 | 0.93 |
| ASQ27 | 0.70 | 0.88 |
| ASQ28 | 1.06 | 0.98 |
| ASQ30 | 0.66 | 0.81 |
| ASQ31 | 0.89 | 1.00 |

*Note:* Noisy or redundant items in bold
